# Analysis of changes in *Betula* pollen season start including the cycle of pollen concentration in atmospheric air

**DOI:** 10.1371/journal.pone.0256466

**Published:** 2021-08-23

**Authors:** Agnieszka Kubik-Komar, Krystyna Piotrowska-Weryszko, Izabela Kuna-Broniowska, Elżbieta Weryszko-Chmielewska, Bogusław Michał Kaszewski

**Affiliations:** 1 Department of Applied Mathematics and Computer Science, University of Life Sciences in Lublin, Lublin, Poland; 2 Department of Botany and Plant Physiology, University of Life Sciences in Lublin, Lublin, Poland; 3 Department of Hydrology and Climatology, Maria Skłodowska-Curie University, Lublin, Poland; Institute for Biological Research, University of Belgrade, SERBIA

## Abstract

Birch belongs to the most important allergenic taxa in Europe, therefore information on the start dates of the pollen season is very important for allergists and their patients as well as for climatologists. The study examined changes in the start of the birch pollen season as well as determined the trend of these changes. Pollen monitoring was performed in Lublin (eastern Poland) in the period 2001–2019 using the volumetric method. The Makra-test was used to detect periods with significantly higher or lower average of the onset than the average for the whole dataset. Two significant falls in the average of the pollen season start were found in 2007 and 2014. Besides, taking into account the 2-3-year rhythm of high and low concentrations of birch pollen in the atmospheric air, linear trends were fitted for the subsets of high and low abundance seasons. Significant changes in *Betula* pollen season start dates were only determined for the highly abundance seasons, while the results for seasons with a low concentration did not allow rejecting the hypothesis about the lack of a linear trend in the changes in the studied parameter. Moreover, a significant polynomial relationship was found between the beginning of a pollen season and the average values of monthly temperatures preceded a season. These analyses show that the start dates of the *Betula* pollen season are getting significantly earlier. The dynamics of changes differ between seasons with high and low concentrations of pollen.

## Introduction

Birch is a common tree in north-western and central Europe [1, 2]. Its pollen is a cause of allergies in the form of allergic rhinoconjunctivitis and probably asthma; disease symptoms in people sensitized to birch pollen appear suddenly, without initial slowly developing symptoms as it is in the case of sensitization to other allergens [3]. The start dates of the birch pollen season differ from year to year [4, 5]. This seasonal characteristic is very important in prevention and treatment of pollen allergy. Allergy symptoms can occur in particularly sensitive people already at a daily concentration of 20 pollen grains/m^3^, whereas in most allergic people they are triggered when the pollen concentration reaches 80 pollen grains/m^3^ [6].

People with allergy to birch pollen account for 6.8% - 57.4% of the population in different European countries [7, 8]. In Poland this group of patients makes up 15% of the population, but allergy cases are found in as many as 21% of children living in cities [9]. Due to allergy to birch pollen, sensitized patients feel a negative impact on health-related quality of life. Different studies reveal significant reductions in emotional well-being and reduction in work performance [8]. In Poland birch pollen reaches very high daily atmospheric concentrations—even 12830 grains/m^3^ [10], with the number of pollen grains in a pollen season being also determined by conditions in the preceding year during the formation of flower buds [11, 12]. In the major part of Poland, the onset of the birch pollen season falls on average between the 10th and 14th of April [5]. However, pollen seasons significantly vary from one another in this respect, mainly due to variable atmospheric conditions and predominantly depending on temperature [13]. It is very difficult to precisely determine the start of the pollen season because of weather conditions that affect it. However, research enriching information on this subject seems to be extremely important from the point of view of people prone to pollen allergy.

Plant flowering and pollen production are very sensitive to thermal stress [14, 15]. There are strong indications that at higher temperatures some plants produce more pollen and bloom earlier [15–17]. Similarly to many tree species flowering in early spring, *Betula* exhibits distinct responses to climate change which are manifested, among others, in an earlier onset of pollen seasons. A clear trend towards an earlier start of the *Betula* pollen season has been found in different parts of Europe: in Denmark [18], in London, Brussels, Zurich, and Vienna [19], in the Netherlands [20], in Switzerland [4, 21], in Reykjavik, Derby, and Sofia [22] as well as in Bavaria [23]. The authors mentioned above found a 10-19-day acceleration of *Betula* pollen seasons during the respective study periods. A study conducted in Poland’s 8 cities over the period 2001–2014 also revealed that the beginnings of the birch pollen season occurred 7–14 days earlier compared to the long-term data [5]. Newnham et al. [24], on the other hand, found no overall change in the onset of the birch pollen season in the UK during a 15-year study period. The studies conducted over the last decade demonstrate that climate change also contributes to an increase in the amount of *Betula* pollen [25]. Moreover, in Germany, an increase in the allergenicity of *Betula* pollen due to the effect of air pollutants such as ozone (O_3_) on pollen grains was found. A positive relationship was shown between the atmospheric ozone levels and the amount of the *Bet v* 1 allergen in pollen grains [26].

This paper presents an analysis of changes in the birch pollen season in Lublin during the period 2001–2019. To this end, we check whether there are statistically significant differences in the onset of the pollen season over the studied period and the trend of these changes. Aditionally, the Makra-test was applied [27, 28] to detect significant breaks in the start date of the birch pollen season and to find out if the start date of the pollen season occurs significantly earlier/later and in which periods of time during years of study (2001–2019).

Furthermore, we investigate the changes in pollen season start, taking into account the biennial cycle of abundant birch pollen production, which is also a novel approach to this topic, similarly to the method of division of seasons in terms of abundance of airborne pollen. For this purpose, we propose an algorithm for dividing the data set in the node of the CART decision tree. Additionally, a model describing the relationship between season start and weather factors is proposed.

## Materials and methods

Pollen monitoring was performed in the period 2001–2019 in Lublin (eastern Poland). A Hirst-type sampler (Lanzoni VPPS 2000) was used for pollen trapping [29]. It was placed in the center of the city on the flat roof of a University of Life Sciences building (22^0^32’25” E and 51^0^14’37” N; 197 m a.s.l.) at a height of 18 m above ground level. Aerobiological investigations were conducted following the recommendations of the International Association for Aerobiology (IAA) and the Quality Control Working Group [30, 31].

The start date of the birch pollen season was determined by the 95% method. This was the day on which 2.5% of the annual pollen integral was recorded. Application of this method eliminates low concentrations of pollen at the beginning and end of the season, which usually originate from long-distance transport or redeposition [32–34]. This method is the most frequently used in aerobiological studies [5, 35–40]. However, other direction of research may be based on the use of different approaches, e.g. cumulative [41], threshold [21], 98% [42], or 90% [32] methods. Ziska et al. [43] propose the use a metric of 4 consecutive days of pollen collection (with the fourth day considered the start of the pollen season).

The following statistical methods were applied in the paper: linear trend fitting, polynomial regression, Makra-test as well as Spearman’s correlation.

The relationship between pollen season start and weather factors was described by a polynomial model. The meteorological data came from the Meteorological Observatory of the Department of Hydrology and Climatology of the Maria Curie-Skłodowska University in Lublin located approximately 1.5 km from the pollen trap. The mean monthly values for temperature (minimum, maximum, mean), humidity, wind speed, and monthly total rainfall were the predictors in this analysis. The values of the weather factors for the period preceding the pollen season were used to build this model. For this reason, the data for April were limited only to the first 10-day period. Due to the high linear relationship between temperature factors, only one type of temperature (minimum, maximum, mean) from a particular month was treated as predictor. The model was built on 18-year data and the year of 2019 was used to check the model stability.

Spearman’s correlation coefficient was used to evaluate the strength of the direct effect of the individual weather parameters included in the derived model on the start date of the birch pollen season.

The Makra-test was used to find significant differences between the averages of all 3,4,5,…,N-1 element subsets of a given time series and the mean of the whole dataset. Its results shows not only when these differences occurred during the studied period but also how long they lasted [28]. The detailed description of the theoretical background for this test as well as examples of its application can be found in Makra et al. [27].

Due to the 2-3-year rhythm of high and low airborne birch pollen concentrations, which is described in the literature [11, 44–46], the linear trend was fitted to the start date values of the pollen seasons divided depending on the level of pollen abundance during the particular season. A division into two subsets was proposed (concentrations: high–H, low–L) based on the decision tree node split implemented in the CART regression trees algorithm [47]. In this procedure, the sum of squares error (SSE) was calculated for each possible splitting of the set consisting of ordered annual total values. The division, which minimalizes SSE was chosen.


$$SSE=\sum_{i\in S_{1}}{\left(y_{i}-{\bar{y}}_{1}\right)}^{2}+\sum_{i\in S_{2}}{\left(y_{i}-{\bar{y}}_{2}\right)}^{2}$$ (1)

In Eq (1), *y*_i_ is the annual pollen integral of a season, while ${\bar{y}}_{1}$ and ${\bar{y}}_{2}$ the mean value of the left- (S_1_) and right-hand side (S_2_) of the possible split (the first and second possible group).

Before proceeding to the analysis, the normal distribution of the data was checked using the Shapiro-Wilk test. This assumption is sufficient to obtain reliable results of the Makra- test. The statistical analysis was conducted using Microsoft Excel and R software.

## Results

**Fig 1:**
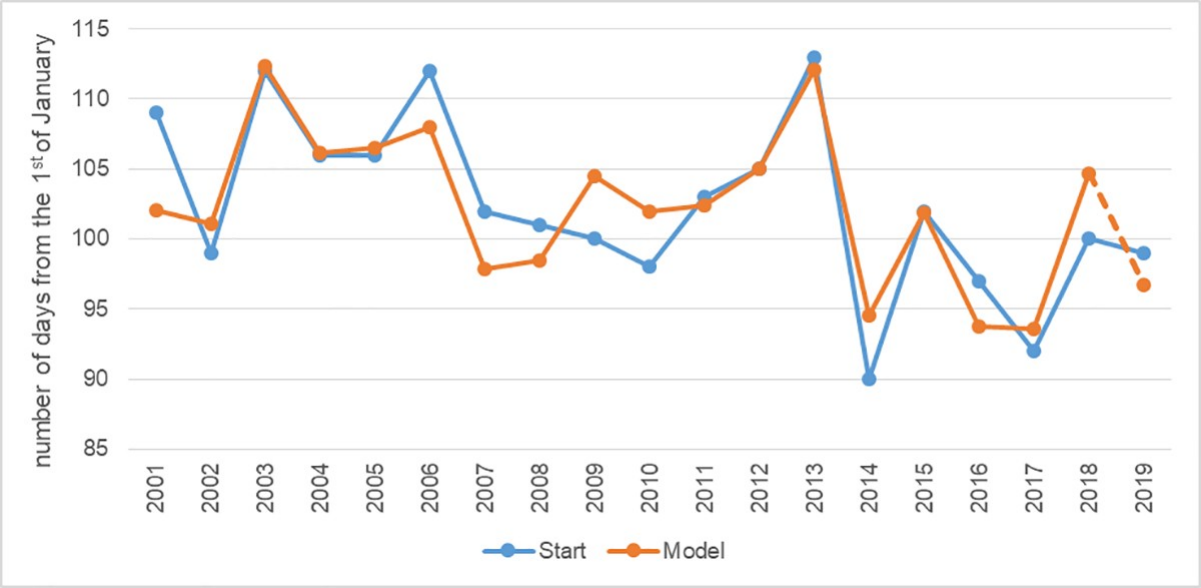
The plot of observed and predicted values of birch pollen season start.

Over the 19-year study period, the start dates of the birch pollen season in Lublin was between March 29 and April 22. The dependence of the Start value on the weather preceding the *Betula* pollen season, i.e. for the period from the beginning of January to the end of the first ten days of April, resulted in finding a model explaining about 74% (R^2^ = 0.738) of the variance in the onset of the birch pollen season in Lublin (Fig 1, Eq (2)).

$$Start=110.5713-1.5201t_{minIII}-0.3026{\left(t_{minIII}\right)}^{2}-1.3199t_{minIV}-0.1016{\left(t_{maxII}\right)}^{2}$$ (2)

where,

*t_maxII_*–mean maximum temperature of February

*t_minIII_*–mean minimum temperature of March

*t_minIV_*–mean minimum temperature of the first 10 days of April

Based on the derived model, it can be concluded that the temperature values for the months preceding the birch pollen season have the greatest effect on the variations in its onset. In particular, taking into consideration the values of the model’s coefficients, it can be stated that these changes are most dependent on minimum March temperature. This analysis is confirmed by the value of Spearman’s coefficient of correlation between the Start parameter and the weather parameters included in the (2) model (Table 1).

**Table 1:** Spearman correlation between model predictors and the start parameter.

Parameter	*t_maxII_*	*t_minIII_*	*t_minIV_*
Start	-0.58*	-0.68*	-0.53*

* Correlation significantly differed from 0 at a 0.05 level

**Fig 2:**
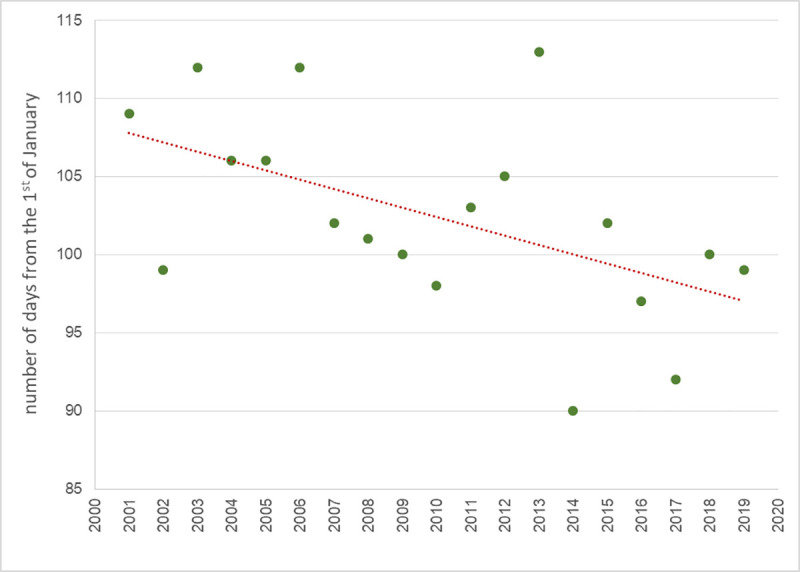
The start of the *Betula* pollen season in Lublin in 2001–2019.

A decreasing linear trend can be fitted in the scatterplot of the Start parameter values, which means an ever-earlier onset of the birch pollen season in Lublin (Fig 2). The regression analysis confirms the occurrence of a significant decreasing linear trend at the adopted level of significance 0.05 (b = -0.596, p = 0.019), however, the degree of its fit to the observation data is poor (R^2^ = 0.28). Thus, determination of variations in pollen season start based on it would carry a large error.

On the basis of the Shapiro-Wilk test results, the hypothesis of a normal distribution of the investigated trait was not rejected (W = 0.96, p = 0.64); thus, the assumptions for Makra-test were met.

The use of the Makra-test in the analysis of changes in the beginning of the pollen season over the 19 research years consists in comparing the mean of all subsets containing from 3 to 18 elements with the overall mean. A total of 152 comparisons were made. Below, we present only statistically significant differences at the adopted level of 0.05 (Table 2). One can observe several levels of fluctuations in the average value of the Start parameter depending on the size of the tested substring. In 2003–2006 and 2014–2017, this difference was the maximum and amounted to about 7 days of difference with the overall average, whereby, the average season started later in 2003–2006 and earlier in 2014–2017 than the average during the 19 research years.

**Table 2:** Differences between the mean start of the subsets and the overall average.

Subset	Period	Difference (rounded)
4-year average	2003–2006*	7
	2014–2017*	-7
5-years average	2003–2007*	5
	2014–2018*	-6
6-year average	2001–2006*	5
	2014–2019**	-6
7- year average	2001–2007*	4
8- year average	2001–2008*	3
11-year average	2003–2013*	3
	2009–2019*	-3
12-year average	2002–2013*	2
	2008–2019*	-2
13- year average	2001–2013**	3
15- year average	2007–2019*	-2

* 0.05 of the significance level

** 0.01 of the significance level

**Fig 3:**
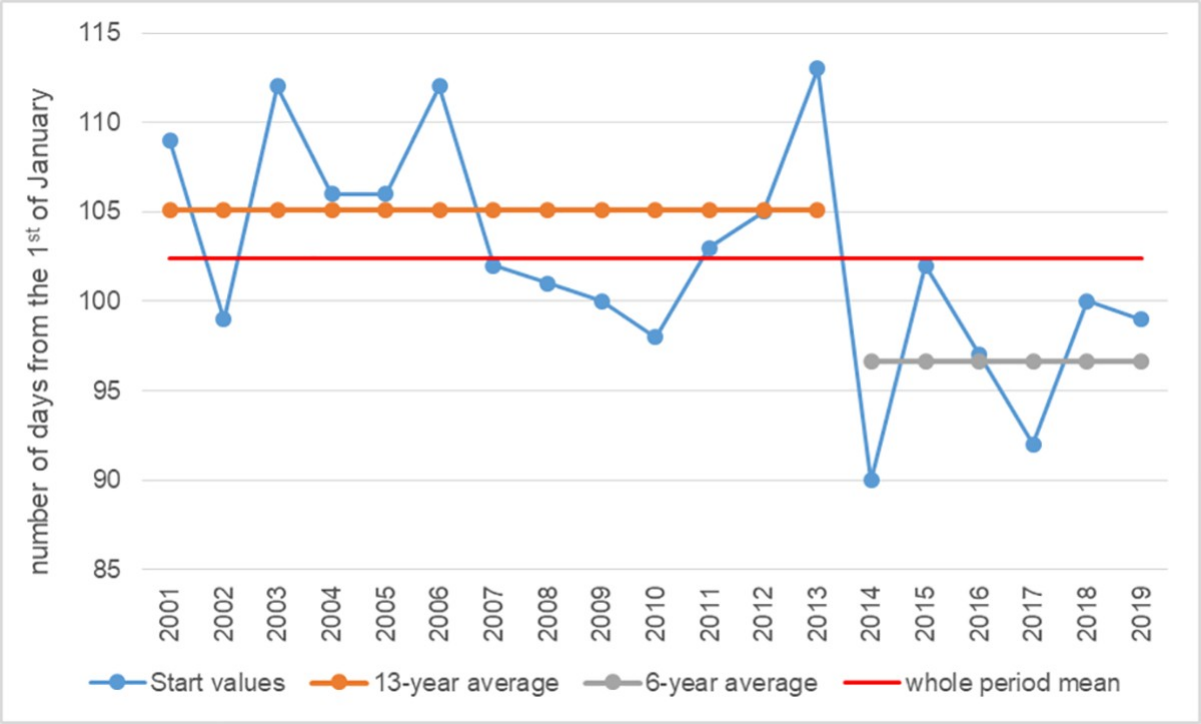
Onset means for sub-periods significantly different from the overall average at a 0.01 significance level.

The division into the two periods: 2001–2013 and 2014–2019 ensured the highest probability of significant differences between these averages and the overall average (the only significant differences at the level of 0.01). The mean start of the pollen season in 2001–2013 was significantly higher than the overall average, while in 2014–2019, it was significantly lower (Fig 3). This means that in the previous 13 research years, the birch pollen season started on average much later than in the following 6 years.

**Fig 4:**
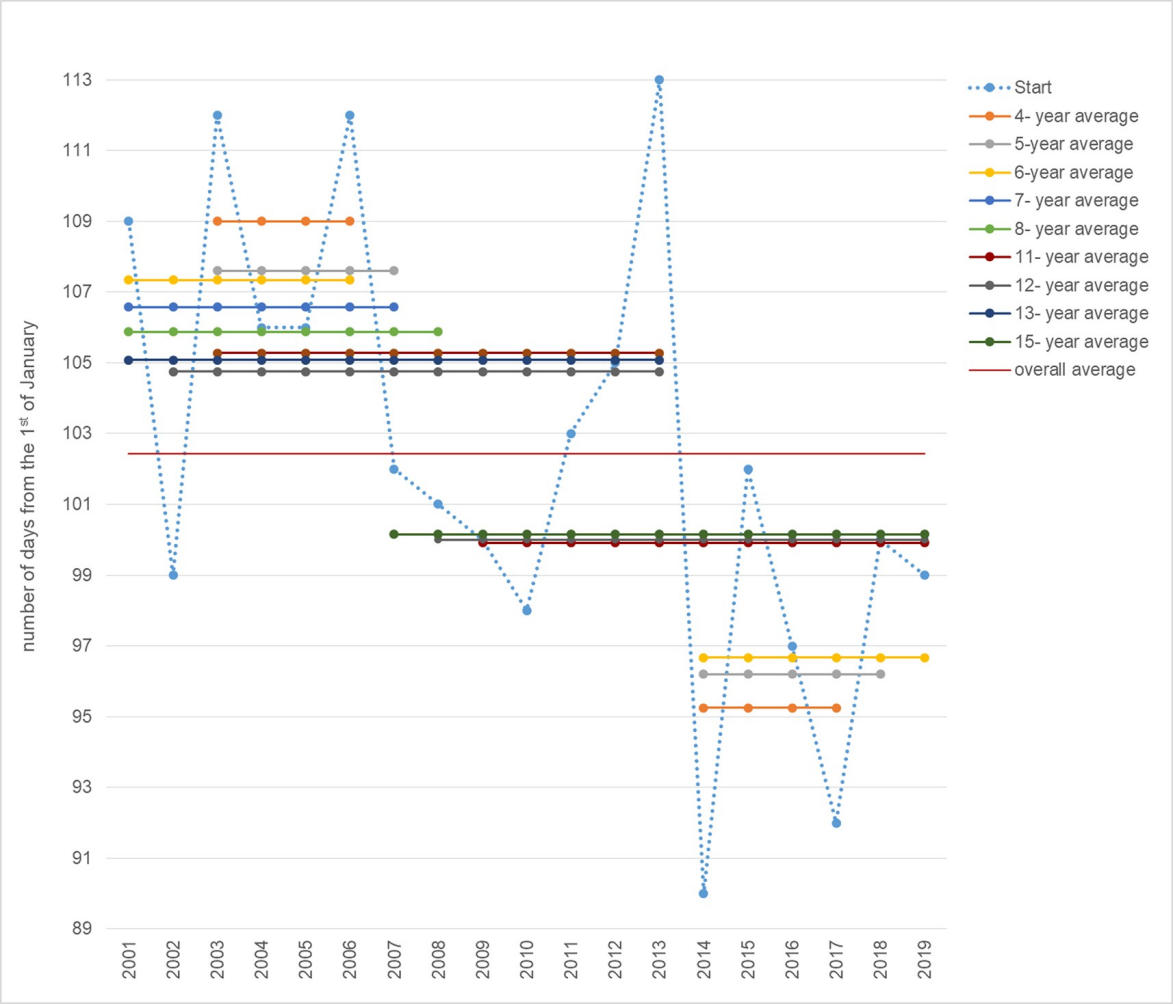
Onset means for sub-periods significantly different from the overall average at a 0.05 significance level.

Based on the data presented in Fig 4, in the period 2001–2013, one can distinguish shorter sub-periods (up to 2008) with a significantly higher average value than the overall average and sub-periods after 2014 with a significantly lower value than the 19-year average. Additionally, clear falls in the mean value of the Start parameter below the general average can be noticed: the first in 2007, from which the average start of the pollen season begins significantly earlier than the average in the 19th year, and the second in 2014, after which the average Start value falls below the 97th day of the year (7.04). It is also worth noting that, after 2014, the start of the pollen season does not exceed the average for the entire data set.

**Fig 5:**
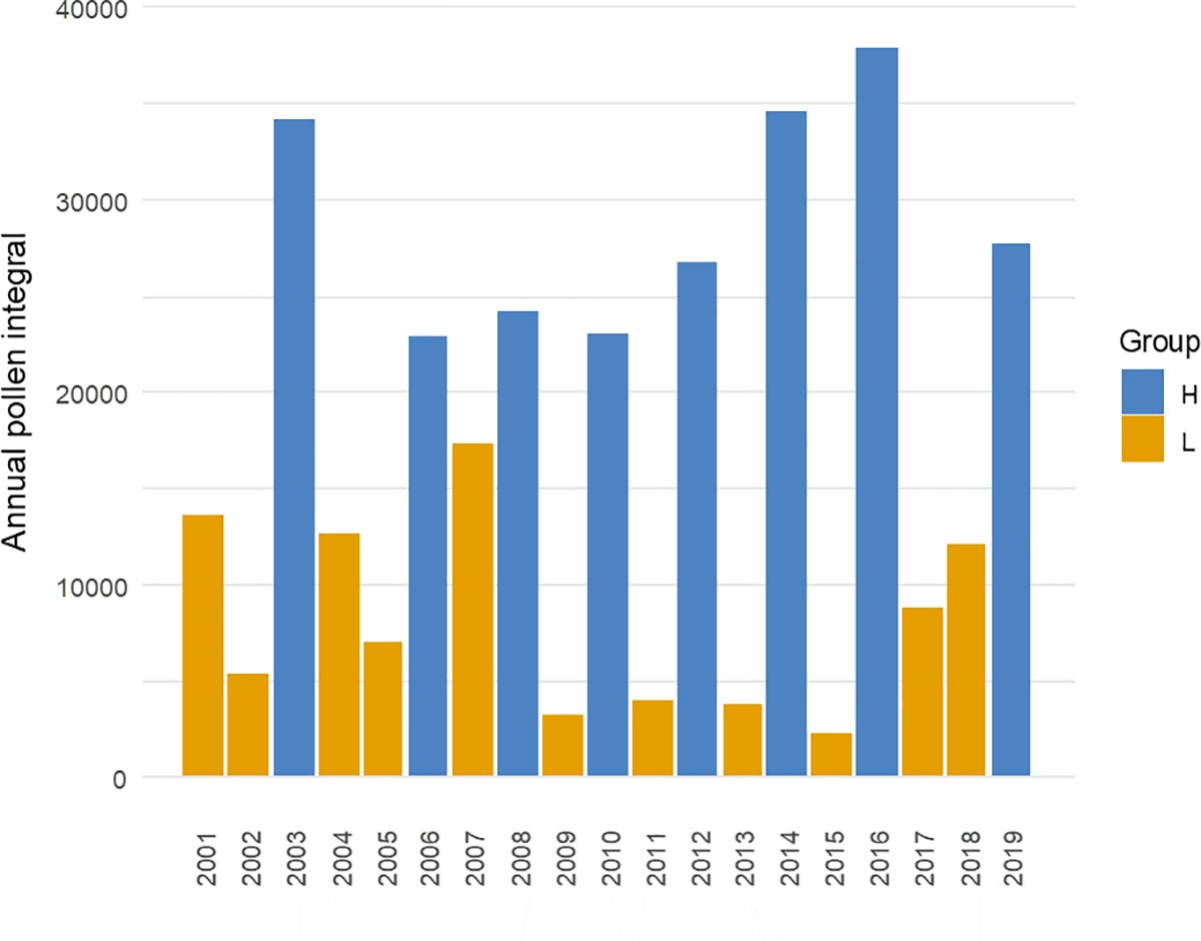
Annual pollen integral for the divided subsets—high concentration (H) and low concentration (L) of *Betula* pollen in atmospheric air.

A previous study on the pattern of birch pollen seasons [46, 48] demonstrated that it can be determined not only by weather factors, but also by trees’ ability to produce male inflorescences in a particular season. Given that, the analysis was deepened by considering two subsets separately–the seasons during which high airborne birch pollen concentrations had been found (H): 2003, 2006, 2008, 2010, 2012, 2014, 2016, 2019, and the seasons with low birch pollen abundance (L): 2001, 2002, 2004, 2005, 2007, 2009, 2011, 2013, 2015, 2017, 2018 (Fig 5). The division was made by minimizing the SSE value (1).

The linear trends were fitted for both data subsets, thereby checking whether a trend towards increasingly earlier onset of pollen seasons in Lublin could be observed both for the abundant seasons and for those with a low concentration of airborne *Betula* pollen.

**Fig 6:**
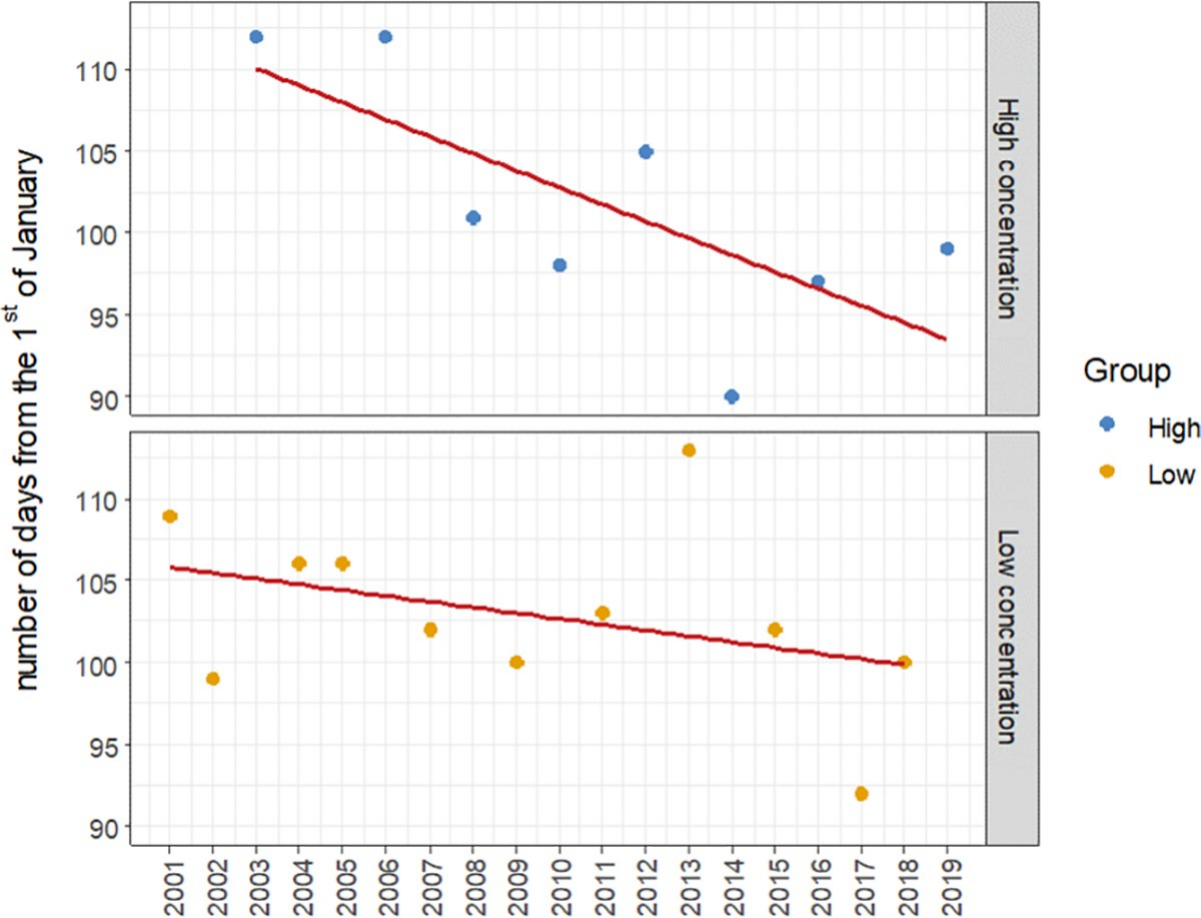
The scatterplot with linear regression line for high concentration (H) and low concentration (L) of *Betula* pollen in atmospheric air.

In Fig 6, we observe a much better fit of the linear trend to the data belonging to group H (R^2^ = 0.52) than in the case of the group L data (R^2^ = 0.14). Furthermore, it is statistically significant only in the case of group H, whereas the gradient of the straight line is almost three times greater, as regards its absolute value (b = -1.035, p = 0.042), than in the case of the data from the less abundant seasons (b = -0.35, p = 0.25). This means that in the years with a higher concentration of airborne birch pollen, the *Betula* pollen season started earlier and earlier, while in the years with a lower airborne pollen concentration no statistically significant trend was found.

We did not decide to build a polynomial model displaying the dependence of the pollen season start on weather conditions for both season types (H, L) due to an insufficient number of observations in the groups in order to provide a reliable model. Nevertheless, using Spearman’s coefficient of correlation, we checked in these groups how strong the relationship is between the Start parameter and the predictors from the (2) model (Table 3).

**Table 3:** Spearman’s coefficient of correlation between model predictors and season start for high concentration (H) and low concentration (L) of *Betula* pollen in atmospheric air.

Season	*t_maxII_*	*t_minIII_*	*t_minIV_*
L	-0.24	-0.53	-0.26
H	-0.75*	-0.78*	-0.77*

* Correlation significantly differed from 0 at a 0.05 level

The values in Table 3 show that there is a much greater, statistically significant dependence between the temperature before the pollen season and the season onset for the abundant seasons. In this case, the minimum March temperature is also most strongly correlated with the investigated characteristic.

## Discussion

This paper shows that the start of the *Betula* pollen season in Lublin in particular years (2001–2019) differs by 23 days between the earliest and latest start dates. Similar results (a difference of 24 days) was obtained in a study conducted in 8 cities of Poland [5]. Large differences in the onset of *Betula* flowering (32 days) were also found in phenological studies carried out in Lithuania over a 30-year study period [49].

Many researchers draw attention to the fact that a biennial rhythm characterized by the alternating occurrence of high and low pollen concentrations is observed in birch pollination [11, 44, 45, 50, 51]. The biennial rhythm of abundant pollen production by *Betula* can also be noted in the results of our study, though over the 19-year study period in Lublin there were sometimes deviations in this cycle since in the years 2001, 2005, and 2018 much lower amounts were recorded instead of high values of annual pollen integrals. The reason for the biennial rhythm can be, among others, adverse meteorological conditions preceding the production of flower buds and subsequently pollen in the years of its low concentrations. This is confirmed by Pidek et al. [52] based on data from the Roztocze National Park as well. Such a possibility is also suggested by the results of studies carried out in 5 cities of Poland over the period 2001–2005, during which the trends in annual pollen integrals were found to be similar to those determined by us in Lublin [10].

Plants are a particularly good measurement tool reflecting the entire set of factors that affect them. In phenological research, different plant growth stages are observed (among others, leaf development, flowering, fruit maturation, leaf abscission). Increasingly greater attention is paid to the importance of the phenology of occurrence of airborne pollen grains of anemogamous plants in relation to global warming. *Betula* strongly responds to air temperature fluctuations, and hence it is a good climate change indicator [53]. A study by Grewling et al. [54] reveals that daily concentrations of *Betula* pollen significantly increased on days with high mean temperatures. In many countries in Europe and the USA, a trend has been observed towards an earlier start of the pollen season in connection with climate change [4, 18, 21, 55, 56]. Moreover, Buters et al. [57] found that temperature has a direct effect on *Betula* allergen release. The observed climate warming is important for public health since allergic people may feel symptoms earlier, and symptoms may get more severe, what may require stronger medication [58, 59]. It follows from the earlier starting and more intensive birch pollen seasons, combined with an increase in the annual mean and peak value of daily pollen concentrations [4, 55].

Previous phenological studies conducted at the regional scale demonstrated that the rate of changes in the beginning of the plant growing season is higher in Western Europe and Scandinavia, and that different phenological rhythms and trends occur in the eastern part of Europe [21, 23, 60–63]. Our aerobiological study confirms the earlier start of the *Betula* pollen season in central Europe and, at the same time, differences in its acceleration in the years with abundant and low pollen production of this taxon. Due to the large variation in pollen abundance, we decided to take this fact into account in conducting the data analysis. The results reveal that the rate of changes, which consist in an ever-earlier onset of the birch pollen season, is more dynamic in the case of the abundant seasons (H) compared to the seasons with low airborne pollen concentrations (L). Thus far, no such relationship that maybe only applies to the above-mentioned part of Europe has been presented. Our study carried out using the variance analysis method and also the trend analysis performed separately for the more and less abundant seasons show the varying rate of changes in tree pollen seasons in a new light.

Our study demonstrates that thermal conditions preceding plant flowering have the greatest impact on the onset of the season. The air temperature in February, March, and at the beginning of April proved to be particularly important. Season 2013, treated as an outliner, was characterized by the lowest minimum temperature in March among the analyzed seasons and a very low minimum temperature in the first 10-day period of April.

The birch pollen season in Lublin began on average on April 11. In some years, the season start was recorded at the beginning of April, but in spite of that we included in the model the meteorological data from the first ten days of April because the low temperature during this period may be associated with a delay in the pollen season. A shorter period (10 years) of the study conducted in Lublin allowed the minimum temperature of February to be indicated as a factor determining changes in the Start indicator [12]. However, an increase in the duration of this study allowed additional determinants to be identified, notably the temperature in March and during the first ten days of April. According to Kasprzyk [53], the timing of birch pollen release strongly depends on the weather in February and March, but the temperature in the first half of March is most important. Newnham et al. [24] found that the start of the birch pollen season in the UK was strongly correlated with mean March temperature.

The Makra-test results indicate a significantly earlier start of the *Betula* pollen seasons in the last 6 years of the study (2014–2019). We found that, after 2014, all pollen seasons were below the average for the entire dataset, which clearly confirms their earlier start. This is probably related to the global warming. The scientific literature shows that the Makra-test has so far been used only in relation to meteorological data [27, 28, 64]. In our research, we showed the possibility of using this test also for aerobiological data.

Another novel approach to this topic used in our research concerns the separate precise statistical analyses performed for the years characterized by abundant and poorer pollen production, respectively. This study showed that in the years of abundant pollen production a more distinct response of studied plants to climate warming in central Poland is observed than in the years with lower pollen concentrations. The determined response of birch is manifested in a significantly earlier onset of birch pollen seasons.

We suppose that this fact is reflected in the overlapping of two factors. On the one hand, when the birch produces more male flowers and the pollen concentration in the air is increased in spring, the season’s start date can be predetermined. On the other hand, in this study, we have shown a strong correlation between the start of the pollen season and the temperature in March, which is increasing due to global warming. It seems that this mutual correlation increases the dynamics of the beginning of pollen seasons in the years with higher pollen concentrations in the air. We also realize that the 19-year study is insufficient for formulation of a strong conclusion in this issue and further investigation is needed.

The weaker response of birch to climate change in the years of less abundant pollen production probably contributes to finding less pronounced average phenological trends in central Poland, similarly to those observed by other authors in eastern Europe [62]. The results of our study are a contribution to the explanation of the different responses of birch, taking into account the biennial rhythm of abundant pollen production.

## Conclusions

These analyses show that the start of the *Betula* pollen season is getting significantly earlier. Its dynamics differ between seasons with high and low concentrations of pollen.

The obtained results demonstrate that the start of the birch pollen season primarily depends on the temperature in the period February–first ten days of April.

The identified reaction of plants is a response to climate warming, which is more evident in the years of abundant pollen production in the alternating cycle of birch pollination. The results of our study reveal the existence of birch response, as regards the phenology of flowering and pollen release, to the changes in climatic conditions in central Europe.

These results also indicate that the start of *Betula* pollen seasons has accelerated over 19 years, especially in the last 6 years (2014–2019).

## Supporting information

S1 Data (PDF)
