# Supplementary material for: Analysis of changes in Betula pollen season start including the cycle of pollen concentration in atmospheric air
Source: PLoS One. 2021 Aug 23;16(8):e0256466. doi: 10.1371/journal.pone.0256466 (PMC8382167; doi:10.1371/journal.pone.0256466)
Supplement: S1 Data — (PDF) [file pone.0256466.s001.pdf]

Table 1 The values of the Start parameter

Year	Start
2001	109
2002	99
2003	112
2004	106
2005	106
2006	112
2007	102
2008	101
2009	100
2010	98
2011	103
2012	105
2013	113
2014	90
2015	102
2016	97
2017	92
2018	100
2019	99

Table 2. Meteorological factors used in the model (2).

Year	t_{maxII}	t_{minIII}	t_{minIV}
2001	2.52	0.14	5.79
2002	6.99	1.48	1.19
2003	-2.09	-0.85	-0.89
2004	1.70	0.67	2.27
2005	-0.55	-3.32	4.33
2006	-1.25	-3.34	3.16
2007	1.54	3.15	3.52
2008	5.61	1.08	5.21
2009	1.61	-0.59	5.02
2010	1.15	0.47	5.82
2011	-0.92	-0.32	6.47

2012	-3.64	1.32	1.29
2013	1.29	-4.54	-0.80
2014	5.74	3.08	3.87
2015	4.35	2.07	1.75
2016	6.60	1.72	6.70
2017	1.85	3.16	6.71
2018	-1.02	-3.11	5.74
2019	6.21	2.31	3.69

t_{maxII} – mean maximum temperature of February

t_{minIII} – mean minimum temperature of March

t_{minIV} – mean minimum temperature of the first 10 days of April
